# Epilepsy educational curriculum for nurses: A report from the Nursing Section of the International League Against Epilepsy

**DOI:** 10.1002/epd2.70273

**Published:** 2026-07-09

**Authors:** Patricia O. Shafer, Sandra Dewar, Ariane Bernier, Ludivine Rohrer, Nancy Santilli, Carmela Redhead, Tammy Gravel, Gagandeep Singh, Jane R. von Gaudecker

**Affiliations:** ^1^ Osborne Health Consulting LLC Wilmington Massachusetts USA; ^2^ Department of Neurology Virginia Commonwealth University Richmond Virginia USA; ^3^ Hospital Department Institution de Lavigny Lavigny Switzerland; ^4^ Neurology Unit University Hospital CHRU Nancy France; ^5^ Santilli Global LLC Sunnyvale California USA; ^6^ Epilepsy Program Vancouver Coastal Health Authority Vancouver British Columbia Canada; ^7^ School of Nursing Massachusetts School of Pharmacy and Health Sciences Boston Massachusetts USA; ^8^ Department of Neurology Dayanand Medical College Ludhiana India; ^9^ School of Nursing University of Virginia Charlottesville Virginia USA

**Keywords:** competencies, curriculum, education, epilepsy self‐management, nursing, training

## Abstract

A comprehensive, competency‐based epilepsy curriculum is needed to strengthen nurses' capacity to care for people with epilepsy worldwide. Such a curriculum should be adaptable across nursing roles, practice settings, and geographic regions. This report describes the development of a nursing curriculum for the International League Against Epilepsy (ILAE). The Curriculum Workgroup of the Nursing Section of the ILAE developed two educational levels: a core level essential for all nurses and a proficient level for nurses who regularly care for people with epilepsy. Each level comprises competencies across five domains: Role of the Nurse, Epilepsy Basics, Nursing Assessment, Nursing Care, and Self‐Management Education and Care. Competencies were evaluated through an online survey assessing agreement using a five‐point Likert scale. Competencies were accepted if ≥60% of respondents rated them as “extremely important.” Items not meeting consensus were reviewed by the Nursing Workgroup and revised, merged, or rejected based on expert opinion. Overall, 55.8% of competencies met acceptance criteria by survey. Remaining competencies were refined through expert review to finalize the curriculum. More than 90% of respondents supported the curriculum, indicated they would use it in practice, and expressed interest in ILAE courses or programs based on the curriculum. This is the first competency‐based epilepsy curriculum for nurses from an international epilepsy organization for worldwide use. It will form the basis of future online and in‐person educational programs for nurses. The curriculum can be adapted to regional contexts worldwide, helping to address gaps in basic nursing education and continuing professional development.

**Key points**

- Enhancing training of nurses, the largest group of healthcare workers, can increase healthcare capacity and improve quality of care for people with epilepsy.
- The Epilepsy Educational Curriculum for Nurses from the ILAE Nursing Section has a tiered approach and can be adapted to different types of nurses, settings, or countries.
- A core level comprises competencies for any nurse, while the proficient level provides additional competencies for nurses who regularly care for people with epilepsy.
- A Self‐Management Education and Care domain provides a unique set of competencies and learning objectives for epilepsy nurse educators.
- Disseminating the curriculum worldwide, integrating it into in‐person and online educational programs, and evaluating outcomes are next steps and challenges.

## 1 INTRODUCTION

The World Health Organization (WHO) highlighted epilepsy as a global public health imperative in 2019 with a mandate to reduce the burden of epilepsy around the world. The global incidence of epilepsy varies by country, with low‐ to middle‐income populations experiencing the highest rates of disease and modifiable risk factors.^1^ As a spectrum disorder, the epilepsies encompass different seizure types, syndromes, etiologies, and comorbidities.^2^ Each can yield different clinical outcomes and challenges. While some epilepsies can be treated by primary care providers, many require specialized care and multiple types of health care professionals (HCP). Unfortunately, specialized epilepsy care is not found in many regions. To address this dilemma, the WHO suggested that a stepped model of epilepsy care be expanded.^3^ This entails educating HCP at the primary care and community level, in specialty settings, and in multidisciplinary teams for care of people with epilepsy. Improving the education and competency of nurses in epilepsy at each level of care is needed to accomplish this.

The WHO Intersectoral Global Action Plan on Epilepsy and Other Neurological Disorders 2022–2031 (IGAP) outlines strategies for improving the health and quality of life for people living with epilepsy and their families.^3^ Strategic objectives of IGAP include: improving access to epilepsy care; providing effective, timely, and responsive diagnosis, treatment and care; and implementing strategies for promotion and prevention of epilepsy and its consequences. As such, these goals have specific implications for nursing practice. Nurses play a pivotal role in the management of epilepsy. As the largest group of HCP worldwide, they are uniquely positioned to potentially bring about meaningful and wide‐ranging improvements in care.^4^ In many regions, nurses serve as frontline providers and may represent the first or sometimes the only HCP encountered by individuals with epilepsy. Their responsibilities span diverse settings, including hospitals, outpatient clinics, community care environments, general medical, and surgical units, as well as specialized epilepsy services.

Nurses contribute substantially across the continuum of epilepsy care. They are integral to the management of acute conditions such as status epilepticus, support patients during diagnostic evaluations and epilepsy surgery, and play a key role in epilepsy monitoring units. Beyond clinical settings, nurses also provide essential care in schools, residential facilities, rehabilitation units, and other community‐based environments. Education of patients and their families constitutes a central component of nursing practice, irrespective of the care setting. Nursing care for people with epilepsy can be inherently holistic and patient‐centered, encompassing not only the diagnostic and therapeutic aspects of disease but also the broader impact of epilepsy on individuals' physical, psychological, and social functioning.^5^, ^6^ Ultimately, nursing practice is directed toward promoting health and well‐being for patients, their families, and the wider community.

A review of treatment gaps in epilepsy identified the need for education of healthcare providers to decrease delays in diagnosis and treatment of seizures.^7^ Further, a needs assessment conducted by the International League Against Epilepsy (ILAE) Nursing Section in 2022 highlighted educational gaps as the greatest barrier for nurses affecting their ability to care for people with epilepsy. Educational needs of nurses may vary due to the diversity of nursing roles, education, and licensure requirements in some areas. For example, advanced practice nursing (APN) roles with prescriptive authority are found more often in high‐income countries as compared to low‐income ones. Manzanares et al. examined differences in APN roles for epilepsy in the United Kingdom and Spain.^8^ APNs in the United Kingdom had more advanced postgraduate education and training, broader knowledge of the APN role for epilepsy, and greater participation in nursing research and disease‐specific conferences than nurses in Spain.

It is widely acknowledged that nurses are disadvantaged by limited training in the neurosciences and an absence of formal epilepsy education. In the United States, the American Association of Neuroscience Nurses (AANN) offers a knowledge‐based certificate program for epilepsy nurses, while the Epilepsy Foundation offers a standardized program for school nurses about epilepsy. In the United Kingdom, the Epilepsy Specialist Nurses Association (ESNA) has recently expanded their role‐based competencies for epilepsy nurses.^9^, ^10^, ^11^ China has developed a competency‐based training content system for new neurology nurses that is not specific to epilepsy.^12^ Unfortunately, no globally relevant competency‐based curriculum on epilepsy is available to address the gaps in nursing education.

While the ILAE has developed curricula tailored for specialists, neurosurgeons, and primary care providers, a comparable tool for nurses was not established until now.^13^, ^14^, ^15^ The development of a consensus‐based, epilepsy curriculum for nurses that can be used worldwide is therefore considered a critical first step toward strengthening epilepsy education for the nursing workforce. The initiative would provide a structured foundation for knowledge and skill development, enhance the quality and consistency of care, and support the integration of nurses more effectively into multidisciplinary epilepsy management worldwide.

In 2018 a nursing task force began examining the literature for nursing roles, impact and educational needs in epilepsy and established the framework for the nursing competencies in epilepsy.^16^ In 2021, the Nursing Section was established and began developing a competency‐based curriculum aligned with ILAE's roadmap for epilepsy education^13^ and incorporating the findings of a needs assessment conducted by the section. The curriculum can be adapted to meet regional needs across the globe, availability and access to resources, as well as different nursing roles. This report presents the curriculum development process, including consensus on a tiered approach to competencies and objectives.

## 2 METHODS

The Curriculum Workgroup consisted of approximately 20 nurses with expertise in epilepsy and/or nursing education. While some changes in membership of this group have occurred over the past 5 years, core members have remained stable. Nurses from five of the six ILAE regions, representing high‐, middle‐, and low‐income countries, participated in the curriculum development. Additional nurses and stakeholders reviewed the curriculum at different phases of development. An integrative approach was used including review of published literature and existing educational programs, expert opinion, and survey evaluation.

### 2.1 Learning domains

The workgroup designed the curriculum for nurses practicing in any setting. The domains were designed to ensure relevance for nurses across all specialties, settings, and practice types. Therefore, they focus on broad foundational knowledge and skills rather than specific topics. The final curriculum domains include Role of Nurse, Epilepsy Basics, Nursing Assessment, Nursing Care, and Self‐Management Education and Care.

### 2.2 Curriculum level

Two levels of curriculum were iteratively developed: core and proficient. The core curriculum was designed for all nurses regardless of their years of experience or the care setting. The proficient curriculum was designed for nurses who regularly provide care to people with epilepsy and other neurological diseases. A proficient nurse would be expected to complete both curriculum levels to ensure comprehensive knowledge and skill development. The frequency and type of care provided by nurses can vary across domains, depending on the type of nurse expertise and setting. For example, a proficient nurse in a hospital setting would manage acute seizures and administer or adjust medications, while an outpatient nurse may focus on chronic care management. A community‐based nurse may provide frontline care in a specific setting (i.e., school, residential or long‐term care, public health setting), manage resources and referrals, and provide public education on epilepsy. And yet, all these types of nurses must understand the use of antiseizure medications and rescue therapies. The core curriculum primarily targets knowledge and comprehension, while the proficient curriculum includes additional dimensions such as application, synthesis, analysis, and evaluation.

### 2.3 Competencies and objectives

Initially the workgroup developed competencies and objectives for the core and proficient levels for each domain. These underwent multiple revisions following review by the workgroup and external experts until consensus was achieved. Terminology for the objectives followed the recommendations from the Einstein Montefiore Center for Professional Development (https://montefioreeinstein.org/patient‐care/for‐professionals/continuing‐medical‐education), a program aimed at improving competence, performance, and patient outcomes for HCP. The survey to evaluate the curriculum was translated to French, Portuguese, and Spanish by bilingual native speakers and examined for ease of use and clarity. Following survey evaluation of the competencies, expert opinion from the Curriculum Workgroup was again used to edit and refine the competencies and objectives.

### 2.4 Evaluation of competencies

An online survey was conducted in English, Spanish, French, and Portuguese from November 2023 through February 2024 to examine agreement with competencies from nurses and other HCP within ILAE, other epilepsy and nursing organizations, and key nursing stakeholders. A convenience sampling approach was used. The survey was disseminated to nurses in the Nursing Section database and was included in ILAE's newsletter, e‐mail, and social media feeds. Regional leaders of the Nursing Section were asked to disseminate it to nurses, nursing groups, and key stakeholders in their regions. The survey was structured to first collect basic demographic and practice information. Respondents were then asked to review and rate each competency using a five‐point Likert scale from “extremely important” to “not at all important.” Respondents could write comments or edits to competencies if desired and submit any areas they believed were missed. They were also asked about their willingness to support and use the curriculum, possible barriers, and if they would recommend an ILAE course based on the competencies.

Descriptive statistics were used to evaluate the ratings by respondents for each item. Competencies were considered to have achieved consensus and were accepted for inclusion in the curriculum if 60% or more of respondents rated an item “extremely important.” Items that received less than 60% endorsement underwent further review and deliberations by the workgroup. Expert opinion and consensus from the workgroup were then used to revise some items for clarity and relevance or to remove them from the curriculum. The tiered curriculum was approved by the ILAE Education Council and the Executive Committee.

## 3 RESULTS

### 3.1 Sample

The survey was completed by 415 respondents, 86.5% of whom responded in English. Respondent demographics were broken down by profession, years of experience, practice setting, location, and World Bank Ranking (WBR) (Table 1). Nurses comprised 83.4% of respondents across all six ILAE regions. The largest group of respondents was from Europe, followed by North America and Asia Oceania. Africa, Latin America, and Eastern Mediterranean regions together accounted for only 17.11% of the sample. Most (79.8%) were from 34 high‐income countries in five regions (Asia Oceania, Eastern Mediterranean, Europe, Latin America, and North America). Twenty respondents (4.8%) were from five low‐income countries in Africa. Urban areas predominated (74.9%) and only 6.8% of the sample were from rural areas. Practice settings for nurse respondents ranged from epilepsy inpatient or outpatient units (46.9%), neurology or neurosurgery units (19.1%), combined neuroscience and epilepsy units (7.8%), general medical or surgical units (6.1%), primary care or community‐based (4.6%), academia (3.5%), critical care and emergency units (2.9%), and school nursing (2.3%). The majority of nurses (69.7%) reported working in epilepsy‐specific settings (center, clinic, or monitoring unit). Respondents reported substantial experience, with 61.7% having 11 or more years in their profession.

**TABLE 1:** Demographics of survey respondents to evaluation of nursing competencies.

	Total *N* ^a^	Number	Percent
Respondents	415		
All Nurses		346	83.4
Physicians		17	4.0
Nursing students		16	3.9
Other HCP		16	3.9
Unspecified/missing^b^		20	4.8
Type of nurse	346		
APNs		159	46.0
Non‐APNs		187	54.0
Nurses who worked in epilepsy center, clinic, monitoring unit	346		
Yes		241	69.7
No		105	30.3
Professional experience (years)	415		
0–10		144	34.7
11–20		118	28.4
>20		138	33.3
Missing/not specified		15	3.6
Location	415		
Urban		311	74.9
Suburban		65	15.7
Rural		28	6.7
Missing/not specified		11	2.7
ILAE region	415		
Europe		180	43.4
North America		99	23.9
Asia Oceania		47	11.3
Africa		36	8.7
Latin America		24	5.8
Eastern Mediterranean		11	2.6
Missing region		18	4.3
Location by World Bank ranking	415		
HIC		331	79.8
LMIC		28	6.7
LIC		20	4.8
UMIC		16	3.9
Missing country		20	4.8

Abbreviations: APNs, advanced practice nurses, including nurse practitioners, clinical nurse specialists, learning disability nurses; HCP, healthcare professional; HIC, High‐income country; LIC, low‐income country; LMIC, low‐middle‐income country; Non‐APNs, registered nurse, licensed practical nurse, registered nurse midwife, community health nurse, public health nurse, nurse scientist, academic nurse educator; UMIC, upper‐middle‐income country.

^a^ Total *N*—415 for all survey respondents; 346 for only nurse respondents.

^b^ Unspecified/missing—7 missing entries, 20 “other” not specified.

### 3.2 Support for curriculum

The survey responses demonstrated robust support for the nursing competencies. Most respondents (94.8%) expressed support for the curriculum to determine the competency of nurses who care for people with epilepsy in their country. A similar response (92.6%) was seen regarding taking or recommending an ILAE course based on these competencies, and 90.4% reported that they would follow the curriculum in their own practice. A total of 299 or 72% of respondents reported 783 barriers. The most frequently cited barriers were cost (24.4%, *N* = 191), time away from work to complete the curriculum (19.3%, *N* = 151), mode of delivery (13.8%, *N* = 108), and access to mentors (12.3%, *N* = 96).

### 3.3 Competency ratings

Based on survey results, 55.8% (*N* = 24) of 43 competencies achieved consensus with ≥60% of respondents rating the items as “extremely important.” Of these items, 16 or 37.2% were kept as competencies, while eight were moved to objectives or merged with another competency. After analyzing responses to open‐ended questions, the workgroup refined the remaining competencies. Of note is that survey respondents had agreed on the majority of core competencies, while some of the proficient items required more deliberation and minor revisions. Fourteen competencies that achieved ratings between 47% and 59% were accepted as competencies after revisions, and two were changed to objectives. Three competencies were excluded from the curriculum based on low consensus scores and expert opinion. These included: working knowledge of epidemiology and etiology (51.4% extremely important), nursing care for nonmedical or alternative treatments (50.9% extremely important), and incorporating EEG into assessment (39.7% extremely important). Content on etiologies is covered in other Epilepsy Basics competencies. Variability in the use of nonmedical or alternative treatments in different regions likely contributed to the lack of consensus. The workgroup felt that this topic could more appropriately be covered as content under treatment and education competencies. While EEGs are a critical part of epilepsy care, most nurses are not involved in the technical aspects of EEG testing. Thus, EEG content can be incorporated under other competencies as appropriate to nursing practice in different parts of the world. Table 2 highlights the top 12 competencies from the survey evaluation. Components of the final nursing curriculum include 30 competencies (20 core and 10 proficient) and 143 objectives (68 core and 75 proficient) (Table 3).

**TABLE 2:** Top 12 nursing competencies from survey evaluation.

Competency	Percent of survey respondents rating item extremely important (%)	Workgroup decision
Describe appropriate first aid for different types of seizures	85.1	Accepted
Know when and how to use rescue medications for seizures	85.0	Merged as objective for medication competency
Demonstrate working knowledge of seizures and epilepsy	81.4	Accepted
Demonstrate working knowledge of seizure recognition and assessment	81.4	Accepted
Demonstrate working knowledge of nursing response to potential and actual seizure emergencies, relevant to scope of practice and setting	80.9	Accepted
Educate people with epilepsy, families/care partners about seizure recognition and first aid	77.7	Accepted
Demonstrate working knowledge of the spectrum of care for people with epilepsy and their families	76.4	Accepted
Identify possible seizure triggers or health conditions that could exacerbate seizures	75.8	Merged as objective for seizure recognition and assessment
Use knowledge of seizure types, postictal phase, and semiology to evaluate safety risks	74.9	Accepted
Educate people with epilepsy, families/care partners on antiseizure medications, adverse events, and potential drug interactions	73.8	Accepted with minor rewording
Demonstrate working knowledge of antiseizure medications, adverse events, and drug interactions for people of different ages and gender	70.0	Accepted
Assess the individual and family for impact of epilepsy on quality of life	69.1	Merged as objective for epilepsy history

**TABLE 3:** Components of epilepsy curriculum for nurses by domain.

#	Domain	Competencies	Objectives
1.0	Role of Nurse	2 (core only)	10 (7 core, 3 proficient)
2.0	Epilepsy Basics	5 (4 core, 1 proficient)	27 (13 core, 14 proficient)
3.0	Nursing Assessment	6 (3 core, 3 proficient)	29 (11 core, 18 proficient)
4.0	Nursing Care	6 (4 core, 2 proficient)	33 (11 core, 22 proficient)
5.0	Self‐Management Education and Care	11 (7 core, 4 proficient)	44 (24 core, 20 proficient)
Total	5 domains	30 Competencies 20 core 10 proficient	143 Learning Objectives 66 core 77 proficient

### 3.4 Final curriculum by domain

Core and proficient competencies and learning objectives by domain are found in Table 4. The first domain, role of nurse, highlights two competencies that focus on how nurses practice, the needs of patients in different regions, and the role of stigma. The domain is not intended to define the nursing role. The Epilepsy Basics domain covers foundational content about epilepsy, such as definitions, causes, seizure classification, diagnosis, and syndromes. The competencies in these two domains received high levels of agreement, while expert opinion streamlined the objectives for the two levels.

**TABLE 4:** Epilepsy curriculum for nurses: domains, competencies, and learning objectives.

Domain	Competency and learning objectives	Level
1.0 Role of Nurse		
1.1	*Demonstrate working knowledge of the spectrum of care for people with epilepsy and their families*	
	1.1.1 Identify regional influences that may affect access to nursing care for people with epilepsy	1
	1.1.2 Identify key aspects of nurse's role in different settings (i.e., primary care, community, hospital, specialty care)	1
	1.1.3 Differentiate nursing care needs of people with uncontrolled seizures compared to those with controlled seizures	1
	1.1.4 Identify health care providers and agencies involved in initial diagnosis and ongoing care of epilepsy/seizures in your region	1
	1.1.5 Describe the role of the nurse on a multidisciplinary epilepsy team	2
	1.1.6 Differentiate key aspects of nurse's role in a specialty or epilepsy specific setting	2
1.2	*Recognize the role of stigma, culture, and family influences on perceptions of epilepsy and barriers to equitable access to care*	
	1.2.1 Identify myths and misconceptions about seizures and epilepsy that may contribute to stigma of epilepsy	1
	1.2.2 Recognize the role of stigma on availability of and access to resources for epilepsy care and services	1
	1.2.3 Analyze how personal beliefs may influence epilepsy care	1
	1.2.4 Explore ways nurses can work to expand access to and increase engagement with epilepsy care and services	2
2.0 Epilepsy Basics		
2.1	*Demonstrate working knowledge of seizures and epilepsy*	
	2.1.1 Identify the criteria to diagnose seizures and epilepsy	1
	2.1.2 Describe the basic pathophysiology of seizures and epilepsy	1
	2.1.3 Categorize common causes of epilepsy according to age in your region	1
	2.1.4 Recognize common causes of provoked and unprovoked seizures among different age groups	2
	2.1.5 Distinguish between simple and complex febrile seizures	2
	2.1.6 Explain criteria for medication‐resistant epilepsy	2
	2.1.7 Identify morbidity risks associated with seizures/epilepsy	2
	2.1.8 Describe major risk factors for sudden unexpected death in epilepsy (SUDEP) and other causes of increased mortality in epilepsy	2
2.2	*Demonstrate working knowledge of nonepileptic events*	
	2.2.1 Identify health problems or conditions that may mimic seizures	1
	2.2.2 Describe common causes of nonepileptic events	1
	2.2.3 Differentiate features of physiological nonepileptic events and functional/dissociative seizures	2
2.3	*Demonstrate working knowledge of how seizures and epilepsy are diagnosed*	
	2.3.1 Describe key aspects of the clinical history and observation that are important for diagnosis of seizures	1
	2.3.2 Identify common tests and tools used in the evaluation of seizures, including potential etiologies	1
	2.3.3 Describe the rationale for and limitations of different types of EEG testing	1
	2.3.4 Discuss the rationale for brain imaging	1
	2.3.5 Discuss patient characteristics that may indicate the need for additional testing to clarify diagnosis, assess treatment response, or localize seizure activity	2
	2.3.6 Identify components of a comprehensive diagnostic workup of patients with drug/medication‐resistant epilepsy and people with complex or rare epilepsy syndromes	2
2.4	*Demonstrate working knowledge of seizure types according to ILAE seizure classification*	
	2.4.1 Describe clinical features of generalized and focal seizures	1
	2.4.2 Differentiate between a focal preserved and focal impaired consciousness seizure	1
	2.4.3 Identify when a seizure may be categorized as having an unknown onset	1
	2.4.4 Describe seizures using the ILAE basic seizure classification	1
	2.4.5 Describe seizures using the ILAE advanced seizure classification	2
	2.4.6 Apply advanced seizure classification to differentiate focal and generalized seizure types	2
	2.4.7 Differentiate major features of the postictal phase of focal and generalized seizures	2
2.5	*Demonstrate working knowledge of epilepsy types and syndromes and their implications for nursing care*	
	2.5.1 Describe common age‐related epilepsy syndromes	2
	2.5.2 Discuss comorbidities that may be associated with common epilepsy syndromes	2
	2.5.3 Apply knowledge of comorbidities to assessment and care of people with specific syndromes	2
3.0 Nursing Assessment		
3.1	*Demonstrate working knowledge of seizure recognition and assessment*	
	3.1.1 Identify type and location of symptoms, responsiveness, consciousness, and duration (semiology) of common seizure types	1
	3.1.2 Differentiate between single, cluster and prolonged seizures, and status epilepticus	1
	3.1.3 Identify possible triggers (lifestyle, environmental, behavioral) or health conditions that could exacerbate or affect seizures	1
	3.1.4 Describe key components of seizure or ictal assessments	2
	3.1.5 Describe EEG changes that may be associated with seizure activity within setting and scope of practice	2
3.2	*Utilize knowledge of seizure types, postictal phase, and seizure semiology to assess safety risks for people with epilepsy*	
	3.2.1 Recognize personal safety risks during a seizure and the postictal phase	1
	3.2.2 Recognize high‐risk activities for people with changes in responsiveness, consciousness, or behavior that may increase risk of injury	1
	3.2.3 Assess potential for postictal psychosis and other behavior changes after seizures	1
	3.2.4 Apply appropriate seizure precautions to care settings and individualize according to risk profiles	1
	3.2.5 Apply knowledge of seizure triggers and comorbidities in assessment of safety risks	1
	3.2.6 Develop plans to minimize risk for postictal psychosis and other behavioral changes after seizures	2
3.3	*Tailor key components of epilepsy history and assessment to different patient populations*	
	3.3.1 Describe common risk factors for development of epilepsy	1
	3.3.2 Incorporate patient and family observations, home recordings, and other self‐reported data into symptom history	1
	3.3.3 Assess the relationship between impact of epilepsy and a person's general health/wellness and quality of life	1
	3.3.4 Incorporate gender‐specific factors in assessment of people with epilepsy across the lifespan	2
	3.3.5 Apply knowledge of aging to assessment of older persons with seizures	2
	3.3.6 Apply knowledge of age and developmental stages into assessment of children and youth with epilepsy	2
	3.3.7 Incorporate knowledge of epilepsy and age‐related differences into planning transitions of care between healthcare providers and settings	2
	3.3.8 Recognize how social determinants of health may impact regional differences in epilepsy	2
3.4	*Demonstrate knowledge of cognitive, mood, and somatic comorbidities in the assessment and care of people with epilepsy*	
	3.4.1 Identify common physical and mental health conditions (comorbidities) that may co‐exist with epilepsy	2
	3.4.2 Recognize ways that physical or emotional events may affect people with epilepsy and the importance of stress management approaches	2
	3.4.3 Incorporate reports of sleep, cognition, mobility, mood, and behavior into epilepsy nursing assessment	2
	3.4.4 Apply knowledge of comorbidities to appropriate screening and referral strategies	2
3.5	*Identify components of assessment and triage of people with new‐onset seizures, appropriate to scope of practice*	
	3.5.1 Describe critical aspects and components of first seizure assessment	2
	3.5.2 Assess safety concerns for people with newly diagnosed seizures/epilepsy	2
	3.5.3 Identify readiness for discussing risks of premature death in persons with epilepsy, as appropriate to setting and nursing role	2
3.6	*Identify critical areas of assessment in people with cognitive disabilities, neurodevelopmental disabilities, and rare epilepsy syndromes*	
	3.6.1 Incorporate reports from family and other care partners in assessment of a person with epilepsy and other disabilities	2
	3.6.2 Recognize impact of neurodevelopmental disabilities on seizures and use of antiseizure medications	2
	3.6.3 Identify ways that epilepsy and disabilities impact a person's ability to undergo diagnostic testing and procedures	2
4.0 Nursing Care		
4.1	*Describe appropriate first aid response to different types of seizures*	
	4.1.1 Describe general principles of care during a seizure	1
	4.1.2 Describe how to tailor first aid to a person during focal, generalized, and unknown seizure types	1
	4.1.3 Identify when and how to call for emergency help during/after a seizure	1
	4.1.4 Recognize types of injuries that may require emergency care	1
4.2	*Demonstrate working knowledge of nursing response to potential and actual seizure emergencies, relevant to scope of practice and setting*	
	4.2.1 Describe seizures based on semiology and differences between seizure clusters, prolonged seizures, and status epilepticus	1
	4.2.2 Recognize when a seizure may become a seizure emergency	1
	4.2.3 Recognize characteristics of convulsive and nonconvulsive status epilepticus	1
	4.2.4 Describe initial nursing care, including airway management, of a person during status epilepticus, relevant to practice setting	2
	4.2.5 Apply understanding of guidelines for managing status epilepticus and institutional/local policies for the administration of antiseizure medications	2
	4.2.6 Discuss the nursing care of a patient requiring continuous EEG monitoring during status epilepticus	2
4.3	*Demonstrate working knowledge of care for patients undergoing diagnostic testing*	
	4.3.1 Apply seizure precautions for people with epilepsy hospitalized for medication changes, EEG monitoring, and presurgical evaluations	1
	4.3.2 Apply knowledge of seizure semiology and purpose of testing to tailor seizure or ictal assessments	2
	4.3.3 Prepare patients for EEG and imaging studies within scope of practice and setting	2
4.4	*Demonstrate working knowledge of antiseizure medications, adverse events, and drug interactions for care of people with seizures of different ages and gender*	
	4.4.1 Describe benefits and risks of treatment with antiseizure medications available in your region	1
	4.4.2 Recognize common adverse effects of antiseizure medications	1
	4.4.3 Identify barriers to taking medications as prescribed for people with epilepsy	1
	4.4.4 Identify factors involved in selection and tolerability of antiseizure medications	2
	4.4.5 Evaluate response to prescribed antiseizure medications	2
	4.4.6 Tailor drug regimens to enhance medication adherence	2
	4.4.7 Apply knowledge of drug interactions to patient safety and management	2
	4.4.8 Identify patients who may benefit from rescue therapy and barriers to their use	2
	4.4.9 Be aware of essential antiseizure medications as suggested by WHO and the potential impact on practice	2
4.5	*Demonstrate working knowledge of treatment options for drug/medication resistant epilepsy*	
	4.5.1 Evaluate the benefits and risks of nondrug options for drug‐resistant epilepsy, including surgery, dietary therapy, and neuromodulation	2
	4.5.2 Provide education and support to people undergoing evaluation and treatment for drug‐resistant epilepsy	2
	4.5.3 Identify special considerations for individuals using dietary therapy for epilepsy	2
	4.5.4 Illustrate monitoring, programming, education, and safety needs for people with neuromodulation devices for epilepsy	2
	4.5.5 Apply pre and postoperative care to people undergoing surgical treatment for epilepsy	2
4.6	*Demonstrate working knowledge of epilepsy care coordination and resource management relevant for setting*	
	4.6.1 Recognize when and how to refer a person to a higher level of epilepsy care	2
	4.6.2 Discuss appropriate resources for people to obtain antiseizure medications	2
	4.6.3 Identify sources of support and education for individuals/families in your region	2
	4.6.4 Provide information and referrals about community resources	2
	4.6.5 Discuss use of the WHO mhGAP Intervention Guide on management of behavioral issues for people with epilepsy in relevant areas	2
	4.6.6 Facilitate collaboration and resource‐sharing among epilepsy/neurology nurses and other nurses from both hospital and community settings	2
5.0 Self‐Management Education and Care		
5.1	*Demonstrate working knowledge of epilepsy self‐management and strategies to individualize care and education*	
	5.1.1 Apply communication strategies that promote engagement, empowerment, and collaboration among people with epilepsy and families	1
	5.1.2 Explore the impact of knowledge and beliefs of seizures/epilepsy on self‐management needs	1
	5.1.3 Assist people with epilepsy and families to identify self‐management priorities and impact on their health and well‐being	1
	5.1.4 Assist people to identify needs for additional supports	1
	5.1.5 Incorporate cultural sensitivity and awareness when educating people about epilepsy	1
	5.1.6 Tailor communication strategies to enhance patient and family engagement, empowerment, and collaboration	2
5.2	*Educate people with epilepsy, families, and care partners about seizure recognition and first aid*	
	5.2.1 Describe how to recognize and assess changes in awareness, behavior, features, pattern, and length	1
	5.2.2 Describe how to keep a person safe during focal and generalized seizures	1
	5.2.3 Ensure that people know how to respond during a seizure	1
	5.2.4 Illustrate when to call for help and when a seizure may be an emergency situation	1
5.3	*Demonstrate working knowledge of seizure action plans and individualizing use of rescue therapies*	
	5.3.1 Demonstrate how to create, use, and maintain seizure action plans	1
	5.3.2 Educate people on the role of rescue therapies in managing seizures	1
	5.3.3 Ensure that people with epilepsy and families have the knowledge, skills, and resources to use rescue therapies safely when prescribed	2
5.4	*Educate people with epilepsy, families, and care partners on use of treatments for epilepsy (i.e., antiseizure medications, dietary therapy, surgery, neuromodulation), adverse events, and adherence strategies within scope of practice*	
	5.4.1 Ensure that people with epilepsy and families have the knowledge, skills, and resources to obtain access to antiseizure medications and other treatments if prescribed	1
	5.42 Ensure that people with epilepsy and families have the knowledge and skills to manage medications, diets, and neuromodulation devices on a daily basis	1
	5.4.3 Counsel people with epilepsy/families on how to recognize and respond to adverse effects and drug interactions	1
	5.4.4 Discuss the importance of adherence to follow‐up care and treatment recommendations with people with epilepsy/families	1
	5.4.5 Tailor adherence strategies to address factors influencing medication, diet, and neuromodulation device management	2
5.5	*Counsel people on strategies for seizure observation and recording, trigger management, and safety management*	
	5.5.1 Describe importance of tracking seizures and triggers to share with family and health care team	1
	5.5.2 Tailor methods of tracking seizures and triggers to capabilities and resources of people with epilepsy and families	1
	5.5.3 Educate people with epilepsy and families on lifestyle modifications to lessen triggers of seizures	1
	5.5.4 Provide guidance on situations in which seizures may result in injury or adverse events	1
	5.5.5 Tailor strategies for safety management to age, seizures, cognitive ability, lifestyle, and settings	2
5.6	*Counsel patients, families, and care partners about SUDEP and other causes of premature death relevant to role and practice setting*	
	5.6.1 Ensure that people with epilepsy and families discuss causes and risk factors for SUDEP and other causes of mortality with their care team	1
	5.6.2 Demonstrate strategies to manage risks for SUDEP and premature death	2
5.7	*Individualize strategies to address psychosocial challenges including education, employment, social aspects, and emotional/mental health*	
	5.7.1 Identify strategies to manage impact of epilepsy on family and social relationships	1
	5.7.2 Counsel people with epilepsy and families on rights and resources affecting education, employment, and discrimination	1
	5.7.3 Assist people with epilepsy and families to identify needs and resources to address social and emotional/mental health challenges	1
	5.7.4 Advocate on behalf of people with epilepsy and families for access to care and resources to manage psychosocial challenges	1
5.8	*Educate people with epilepsy, families/care partners about treatment options for nonepileptic events*	
	5.8.1 Ensure that people with epilepsy and families have plans for recognizing, responding to, and treating nonepileptic/functional dissociative events	2
	5.8.2 Assist people with epilepsy/families to identify needs and resources for managing comorbidities and the consequences associated with nonepileptic events	2
5.9	*Counsel people with epilepsy and their families/care partners on strategies for managing drug‐resistant epilepsy*	
	5.9.1 Ensure that people with epilepsy/families can describe the causes and consequences of uncontrolled seizures	2
	5.9.2 Facilitate discussion of benefits and risks of treatment for drug‐resistant epilepsy	2
	5.9.3 Develop plans for managing and navigating treatment process	2
	5.9.4 Tailor strategies to support emotional health of people with epilepsy and families during and after the treatment process	2
5.10	*Apply knowledge of cognitive and behavioral disabilities when educating people with epilepsy, families/care partners*	
	5.10.1 Recognize cognitive and behavioral factors affecting person's ability to manage their epilepsy	2
	5.10.2 Assist people with epilepsy/families to develop plans and identify resources for managing comorbidities including impact on seizures/epilepsy	2
	5.10.3 Tailor plans for managing and navigating self‐management needs	2
	5.10.4 Advocate for people with epilepsy and families to access health care and community resources to assist with self‐management and independent living needs	2
5.11	*Counsel people with epilepsy, families/care partners about hormonal issues, reproductive health, and parenting throughout the lifespan*	
	5.11.1 Describe the bidirectional relationship between hormones and epilepsy	2
	5.11.2 Identify impact of epilepsy and its treatment on hormonal function and reproductive challenges	2
	5.11.3 Tailor plans to maximize seizure control and lessen risks for people of child‐bearing potential including during and after pregnancy	2
	5.11.4 Tailor self‐management education and resources to support breastfeeding, safe parenting, and emotional health	2
	5.11.5 Advocate for people with epilepsy and families to access appropriate care for reproductive health and parenting	2

*Note*: Level 1 = Core level. Level 2 = Proficient level.

Domains 3 and 4 focus on skills that nurses need regardless of practice setting. Nursing Assessment emphasizes areas that may contribute to different types of care. For example, recognizing and assessing what happens during a seizure is important for seizure first aid, developing seizure action plans, treating acute seizures, keeping a person safe, and preparing patients for surgical evaluations. Seizure assessments at the core level focus on knowing what to observe, recognizing triggers, and differentiating seizure patterns (i.e., single, cluster, prolonged seizures). However, the proficient nurse should be able to conduct a more interactive seizure assessment to identify changes in responsiveness, cognition, behavior, or movement. Unique areas of assessment for different populations are highlighted and covered in more depth at the proficient level. For this domain, a nurse's practice setting will dictate how the findings are used.

The Nursing Care domain builds on the assessment skills and addresses basic care needs for people with epilepsy (i.e., seizure first aid and safety, responding to emergencies, managing medications, care for hospitalized patients). While proficient objectives are identified for almost all care competencies, care of patients with drug‐resistant epilepsy, care coordination, and resource management are unique to the proficient nursing curriculum.

The Self‐Management Education and Care domain encompasses the knowledge, skills, and resources that patients and families need to manage their epilepsy.^2^ While many disciplines contribute to education of patients and families, nurses consider this aspect fundamental to their practice. Although group education can be beneficial in certain contexts, nurses focus on individualizing and tailoring education to meet the specific needs of each patient. These competencies focus on the role and responsibilities of nurses, rather than the patient outcomes associated with epilepsy self‐management. A comprehensive group of competencies is included in this domain, such as educational needs for any person with epilepsy (i.e., seizure recognition and first aid, safety, tracking seizures and managing triggers, using seizure action plans and rescue therapies) and those that focus on treatments, mortality, comorbidities, psychosocial challenges, and women's health. It is incumbent upon the healthcare team to help patients and families identify their needs and ensure that these needs are met.

## 4 DISCUSSION

This epilepsy curriculum for nurses, developed with survey consensus and expert opinion from the Curriculum Workgroup, offers a framework that can be used and tailored to any nurse or practice setting. The core curriculum will fill gaps in basic nursing education and can be used by practicing nurses worldwide. The proficient level will provide guidance for nurses specializing in epilepsy and related fields, addressing the knowledge and skills that are developed through informal or “on‐the‐job” training. This curriculum will be a living document, evolving as our knowledge about epilepsy, its treatment, and nursing practice grows.

The majority of competencies in this curriculum achieved consensus from respondents to the survey; however, it is interesting to note the lower agreement for some of the proficient competencies. Initially, curriculum working group members anticipated differences among types of nurses. However, further analysis of survey responses found more differences among ILAE regions than between advanced practice nurses and other nurses.^17^ There are many factors that could contribute to this finding, including the reach of survey dissemination in some regions (Africa, Eastern Mediterranean, and Latin America), a low number of responses from low‐income countries and rural areas, and available languages for the survey. Yet nursing practice and the availability of epilepsy care have regional variations as well. Regions with limited epilepsy specialists or comprehensive epilepsy centers will have fewer nurses proficient in epilepsy, potentially affecting evaluation of these competencies. Also, some of the proficient competencies are geared to care of specific groups of patients with epilepsy or topics not encountered in all settings. These factors underscore the need for standardized training of nurses and for flexibility and tailoring of the curriculum across regions.

This curriculum does not offer a specific pathway for advanced practice nurses specializing in epilepsy. However, completion of the core and proficient nursing levels, along with select epileptology competencies, may fulfill needs of advanced practice nurses working in epilepsy. This nursing curriculum is not intended to replace country‐specific programs or role competencies. However, it can fill gaps for those countries, schools of nursing, and professional education organizations and increase the capacity of nurses able to provide care to people with epilepsy.

The concept of self‐management has been a longstanding part of nursing care. However, the application of self‐management and patient/family education in the context of epilepsy has varied considerably. While several evidence‐based programs have been developed, many are focused on managing comorbidities of epilepsy and have demonstrated limited long‐term impact on seizure control or quality of life.^18^, ^19^, ^20^ To comprehensively address multifaceted aspects of epilepsy, self‐management has been systematically organized into 11 competencies under the Self‐Management Education and Care domain. This framework is designed to emphasize the goal of helping patients and families effectively manage epilepsy and overall health. By emphasizing the nurse's role in individualizing self‐management education, these competencies can serve as the basis for the epilepsy nurse educator role.

The barriers to use of the curriculum were expected and include cost, time to complete, and method of delivery. Unfortunately, these barriers are common in nursing education because nurses are usually not compensated in terms of time or funds to attend or participate in professional education. Thus, any nursing education program needs to consider these factors as well as regional differences and the ability to provide low‐cost or remote programming. Webinars and telementoring programs may overcome these barriers and allow adaptability among countries.

### 4.1 Limitations

Fewer responses from low‐income countries were received which may affect the generalizability to nurses in some countries. However, the curriculum can be tailored to the needs of different countries and types of nurses. For example, countries with no specialized epilepsy care, access to surgery or neuromodulation could choose not to use those competencies.

## 5 NEXT STEPS

1. The curriculum will be disseminated to all ILAE member countries for use and published on the ILAE website. Dissemination to regional professional nursing organizations, schools of nursing, and relevant stakeholders is encouraged. This initiative will standardize care practices, provide a comprehensive approach to epilepsy management, and improve patient outcomes through consistent, evidence‐based training.
2. Translation of the curriculum into multiple languages will help dissemination and implementation, as English is not a common language for nursing education globally.
3. The Nursing Section is already implementing the curriculum into in‐person and virtual educational programming. The development of educational modules based on this curriculum should be conducted in multiple languages and hosted on web‐based learning platforms. These platforms must effectively address barriers nurses often encounter. The ILAE Academy exemplifies such a platform.
4. It will be critical to assess the impact of using this competency‐based curriculum. Program evaluations can offer insight into perceived knowledge and self‐efficacy of nurses. For example, in the United States knowledge about epilepsy and self‐confidence improved after nurses completed the AANN epilepsy certificate program^21^ and after school nurses participated in a telementoring program in the United States.^22^ Ideally, nursing education would have broader impacts on patient care. A nursing intervention in the United Kingdom for adults with intellectual delays and epilepsy found that a competency‐based framework had the potential to reduce the cost of care delivery.^23^ Further research is encouraged to examine the impact of nursing education, using this ILAE competency‐based curriculum, on nursing practice, patient outcomes, and quality of care.

## 6 SIGNIFICANCE

As the largest healthcare workforce globally, nurses play a pivotal role in improving patient care outcomes. It is critical that professional nurses have the competency to care for people with seizures and their families. Training nurses in epilepsy care and management can significantly improve outcomes and increase capacity of the healthcare workforce. As a living document, the epilepsy curriculum for nurses will provide a framework to move forward and enhance professional nursing practice. Investing in education and resources for nurses in epilepsy care is crucial to advancing healthcare delivery, reducing the treatment gap, and promoting health and wellness for patients, families, and communities.

## SUPPLEMENTAL MATERIAL

The entire curriculum is available at https://www.ilae.org/.

**Test yourself**

1. The Epilepsy Curriculum for Nurses is intended only for nurses who specialize in epilepsy.
  - True
  - False
2. Which of the following is not a reasonable learning objective for nurses using the core curriculum?
  - Differentiate nursing care needs of people with uncontrolled seizures compared to those with controlled seizures
  - Describe seizures using the ILAE basic seizure classification
  - Describe how to tailor first aid to a person during focal, generalized, and unknown seizure types
  - Discuss nursing care of a patient requiring continuous EEG monitoring during status epilepticus
3. Which of the following statements about the Self‐Management Education and Care domain is true?
  - It provides core competencies and learning objectives for nurses to help patients and families manage epilepsy
  - It cannot be adapted to regional needs and priorities
  - It provides outcomes for patients and families on how to manage epilepsy
  - Nurses must work in an epilepsy‐specific setting to use these competencies
*Answers may be found in the* supporting information.

## Supporting information


Appendix S1.

## Data Availability

The data that support the findings of this study are available on request from the corresponding author. The data are not publicly available due to privacy or ethical restrictions.
