# Supplementary material for: Epilepsy educational curriculum for nurses: A report from the Nursing Section of the International League Against Epilepsy
Source: Epileptic Disord. 2026 Jul 9;28(5):1359–73. doi: 10.1002/epd2.70273 (PMC13695475; doi:10.1002/epd2.70273)
Supplement: Supplementary file 1 [file EPD2-28-1359-s001.docx]

**TEST YOURSELF**

**Answers**

**1. B**

**2. D**

**3. A**
